# OD15 Multiparametric Magnetic Resonance Imaging In Biopsy-Naive Patients: A Real-World Evidence For Decision-Making In Prostate Cancer Pathway

**DOI:** 10.1017/S0266462324001491

**Published:** 2025-01-07

**Authors:** Genevieve Asselin, Melanie Tremblay-Boily, Yves Fradet, Marc Rhainds

## Abstract

**Introduction:**

Use of multiparametric magnetic resonance imaging (mpMRI) in the prostate cancer (PCa) diagnostic pathway could reduce prostate biopsy (Bx) in Bx-naive patients. The objective was to assess the feasibility of implementing a new PCa diagnostic pathway with the addition of mpMRI in a real care environment.

**Methods:**

Following an HTA report published in 2019 by our team, a committee involving stakeholders (e.g., urologists, radiologists, hospital managers) was created to review the PCa diagnostic pathway including mpMRI in Bx-naive patients and both systematic and targeted 3DTRUS-MRI fusion Bx when Bx was recommended. Data in the new PCa diagnostic pathway were collected between September 2021 and June 2022. The comparison group is a cohort of 629 men who underwent an initial systematic transrectal ultrasound Bx in 2017 when prostate mpMRI was not available. Clinically significant PCa (csPCa) was defined as Grade Group ≥2.

**Results:**

In 2021 and 2022, 1,336 Bx-naive patients were referred to a urologist. Recommendations were: 703 (53%) for follow-up in six to 12 months, 254 (19%) directly to systematic Bx, and 379 (28%) to mpMRI. Overall, csPCa was diagnosed in 246/427 (58%) patients referred to mpMRI or Bx in the 2021 to 2022 cohort compared to 274/629 (44%) patients in the 2017 cohort (p<0.05). The new diagnostic pathway prevented 33 percent of patients from having Bx. Shorter delays between initial consultation with urologists and transmission of Bx results were observed for patients referred directly for prostate Bx compared to mpMRI before Bx (mean: 2.8 vs 9.1 months).

**Conclusions:**

Experimentation in a real care setting has highlighted the added value of the early involvement of urologists in the diagnostic pathway of prostate cancer for the triage of patients. Integration of mpMRI was associated with a lower number of patients referred for prostate Bx and a higher csPCa detection rate.

